# Not every classroom lifts you up: school academic emphasis, teacher encouragement and motivation, and student constraints in reading achievement—a multilevel latent profile analysis

**DOI:** 10.3389/fpsyg.2026.1680413

**Published:** 2026-01-27

**Authors:** Selçuk Turan

**Affiliations:** Ereğli Faculty of Education, Department of Educational Sciences, Zonguldak Bülent Ecevit University, Zonguldak, Türkiye

**Keywords:** classroom climate, classroom instruction, classroom instruction limited by student attributes, multilevel latent profile analysis, school emphasis on academic success, student reading achievement, teacher encouragement and motivation

## Abstract

**Introduction:**

This study aims to identify latent profiles that emphasize academic success and examine how these profiles affect the interaction between classroom reading instruction, classroom climate, and student reading achievement.

**Methods:**

Employing multilevel latent profile analysis combined with a cross-level moderation approach, the study used PIRLS 2021 data from 6,032 students across 192 schools in Türkiye.

**Results:**

The findings revealed two school profiles as perceived by teachers, one with a low emphasis on academic achievement and the other with a high emphasis. More specifically, in schools where teachers perceived a stronger focus on academic success, their encouragement and motivation efforts were more linked to higher student reading achievement. In contrast, in schools with a low emphasis on academic success, classroom instruction limited by student attributes negatively affected reading achievement; however, this association remained consistent across both profiles, indicating that school emphasis did not moderate this relationship.

**Discussion:**

This study contributes theoretically by extending the School Effectiveness framework to show that school academic culture shapes the impact of classroom practices. The findings refine this framework by illustrating that instructional effects differ across schools with varying levels of academic emphasis. These results underscore the need for policies and leadership practices that cultivate strong academic cultures to enhance the effectiveness of classroom instruction.

## Introduction

Improving student achievement has become a global concern, especially in education systems shaped by increasing accountability and the demands of knowledge-based economies (OECD, 2019a; UNESCO, 2017). A huge body of research suggests that student achievement may depend on a variety of factors, such as family background (Coleman et al., 1966; McCoach et al., 2010), school leadership (Heck and Hallinger, 2010; Özdemir, 2019; Özdemir et al., 2024; Sun and Leithwood, 2012), instructional quality (Kyriakides et al., 2013; Toropova et al., 2019), and school-based factors (Teodorović, 2011). Among these school-based factors, school emphasis on academic success (SEAS) represents a core element of the institutional climate, including high academic expectations, effective curriculum implementation, collaboration among teachers, and parental involvement. For schools, this emphasis signals organizational values and collective responsibility for achievement, while at the teacher level it is reflected in classroom practices such as setting high expectations, providing motivational support, and structuring instruction effectively (Mullis and Martin, 2019). Empirical evidence has demonstrated a strong, positive association between focusing on academic success and improved student achievement (Martin et al., 2013; Nilsen and Gustafsson, 2014). However, relatively fewer studies have examined how the broader school culture, specifically teachers’ collective emphasis on academic success, shapes the impact of classroom-level practices on student learning outcomes (Lee and Smith, 1999; MacNeil et al., 2009; McChesney and Cross, 2023). This reveals a critical gap in understanding how school-wide values and expectations may condition the effectiveness of instruction and climate.

While a growing body of previous research has highlighted the role of SEAS in shaping student achievement (Ceylan and Sever, 2020; Nilsen and Gustafsson, 2014; Ye et al., 2024), there are still several key areas that require further investigation. First, much of the existing literature is designed to be compatible with the decentralized governance structures of some western societies. By contrast, Türkiye operates within a long-standing centralized system, where key decisions are made at the national level. Examining SEAS in this context may offer insights relevant to similar centralized systems. Second, since many studies focus on variable-centered approaches, there are relatively few studies on person-centered approaches (Özdemir et al., 2024). However, person-centered approaches that take into account the unique characteristics of schools and teachers are equally important (Ryan and Deci, 2000). This study aims to fill this gap by adopting a person-centered approach to discover different school profiles based on the importance they place on academic achievement. Finally, one question that remains unclear in the literature is how a collective emphasis on academic success among teachers can influence various educational outcomes (Gustafsson et al., 2018). The aim of this study is to address this issue by examining how emphasizing academic success profiles affects the relationship between classroom climate and student achievement.

The potential contribution of this study is to demonstrate the applicability of findings based on data obtained from centralized education systems, such as Türkiye’s, to other countries with similar structures. In such systems, where hierarchical decisions are predominantly determined by central authorities, SEAS reflects shared expectations and collective norms that shape teachers’ motivation, instructional choices, and classroom organization. As a school-level cultural feature, SEAS profiles capture meaningful variation in these norms and can amplify or constrain the extent to which classroom practices translate into student outcomes, thereby functioning as theoretically grounded cross-level moderators within the study’s framework. Building on this rationale, the research questions are as follows:

*RQ1*: What distinct profiles of schools can be identified based on their emphasis on academic success, according to teacher perceptions?

*RQ2*: Do school profiles moderate the relationship between classroom reading instruction, classroom climate, and student reading achievement?

### Turkish context

The Turkish education system is characterized by a highly centralized and hierarchical structure, with the Ministry of National Education (MoNE) dominating managerial processes, including curriculum development, personnel assignment, and budgeting (Bellibaş et al., 2021; Özdemir et al., 2023b). Teachers are seen as the mere implementers of the curriculum and policies determined by MoNE within such a highly centralized system (Bektaş et al., 2022; Recepoğlu and Kılınç, 2014), in which they have limited autonomy in classroom teaching practices (Şahin et al., 2017).

Over the last quarter of a century, MoNE has focused on improving the quality of teaching and learning in schools through structural reforms. The adoption of the constructivist teaching approach in 2005 marked a shift from a teacher-centered to a student-centered model (Ministry of National Education [MoNE], 2005). In 2017, MoNE published a “Teacher Strategy Document” outlining general competencies for the teaching profession and key policies for teacher development [Ministry of National Education [MoNE], 2017]. Finally, MoNE implemented the “Teaching Profession Law (2024),” defining career steps for teachers, including the specialist and head teacher titles based on a central examination (Official Gazette, 2024).

## Literature review

### School emphasis on academic success

Despite variations in how researchers conceptualize the construct, academic emphasis is consistently portrayed in the literature as a multidimensional feature of the school environment that reflects shared attitudes and expectations toward learning. This concept involves several factors, including commitment to high academic standards, establishing expectations that encourage students to do their best, and leadership regarding improvements in student achievement by teachers and principals (Goddard et al., 2000). Moreover, it covers the emphasis on social relationships that center around academic pursuits (Wang and Degol, 2016). Elaborating further on this model, Martin et al. (2013) proposed the TIMSS construct labeled SEAS, characterized by the existence of challenging curricular goals, qualified teachers who are competent to deliver, students motivated to achieve, and parents who offer support for learning.

The literature has indicated that academic emphasis is closely related to collective efficacy, which enhances academic achievement and builds trust among the members of the school community, parents, and students. Although these two concepts are interrelated, academic emphasis is an external environmental factor that directs students toward success, while collective efficacy affects academic achievement through different mechanisms as an internal psychological belief that increases teachers’ motivation and the effectiveness of teaching processes (Hoy et al., 2002). In turn, all these components create a supportive academic environment fostered by academic optimism, which is a school property (Hoy et al., 2006; McGuigan and Hoy, 2006; Wu et al., 2013). Academic emphasis is also considered an important sub-dimension of school climate. This emphasis reflects the shared values and expectations of the school community, while shaping not only students’ academic but also their social and behavioral experiences (Thapa et al., 2013; Rudasill et al., 2018). Therefore, academic emphasis is a fundamental component representing the academic dimension of school climate. Since PIRLS 2011, the SEAS scale has allowed principals and teachers to describe their schools according to attitudes and behaviors from teachers, parents, and students that are supportive of academic success. In this regard, the scale provides a means to examine how variations between schools relate to differences in students reading achievement (SRA) in large-scale assessments.

### Teachers encourage and motivate students

Teachers play a critical role in motivating students to read, which is fundamental for their reading development (Cambria and Guthrie, 2010). This central role aligns with broader research positioning teacher practices as foundational to students’ motivation and engagement in reading. By acting as motivational role models and creating book-rich environments, teachers can greatly impact children’s reading habits (Gambrell, 1996). Motivation is a key factor in reading engagement; motivated students set more reading goals, value their reading abilities, and have confidence in their skills, leading to greater involvement in reading activities (Barber and Klauda, 2020). *Teachers encourage and motivate students* (TEMS) can be defined as the ways in which teachers foster student motivation by promoting autonomy, such as by allowing students to choose their reading materials, and by building supportive teacher-student relationships that enhance the learning environment (Mullis and Martin, 2019). This theoretical perspective is strongly supported by empirical research, as numerous studies have shown that teachers’ ways of encouraging students, particularly by providing emotional support, granting students autonomy, and giving motivational feedback increase students’ academic participation and success (Reeve, 2006; Wentzel, 1998; Skinner and Belmont, 1993). Empirical evidence consistently shows that motivationally supportive teacher behaviors foster both engagement and achievement. The support students receive from their teachers can help them develop more positive attitudes toward learning, exert more effort, and achieve greater success, particularly in basic skills such as reading (Guthrie et al., 2004). In autonomy-supportive classroom environments, students’ motivation and participation in class increase significantly, which directly reflects on academic performance. Also, providing students with the autonomy to select their reading materials is particularly impactful in enhancing their motivation (Gambrell, 1996). Additionally, teachers’ supportive and empathetic behavior, combined with other motivational strategies, is crucial in promoting reading motivation and engagement, ultimately fostering a positive and productive learning environment (Cornelius-White, 2007; Monteiro, 2013). Overall, existing research positions teachers’ encouragement and motivational behaviors as key drivers of students’ reading motivation.

### Classroom instruction limited by student attributes

Classroom climate plays a foundational role in influencing students’ learning experiences, and the quality of instruction greatly depends on different characteristics of the students, such as readiness, physical readiness, mental readiness, cognitive readiness or physiological readiness (Mullis and Martin, 2019). These dimensions collectively illustrate how student attributes form an integral part of the classroom climate. Student-teacher relationships, the social atmosphere in the classroom, the structure of teaching processes and students’ attitudes toward academic achievement are the main components of classroom climate (Fraser, 1998). Pianta et al. (2008) emphasize that classroom climate is determined by factors such as teacher support, student engagement and classroom management and has a direct impact on academic achievement. Moreover, Bronfenbrenner (1979) defines the classroom environment as a microsystem in student development and explains how the interactions between students and teachers shape academic achievement. Together, these perspectives frame the classroom as a dynamic setting in which instructional quality is closely tied to student characteristics. In this context, a positive classroom climate creates a foundation that supports the effectiveness of teaching processes while increasing students’ academic motivation and engagement (Wang and Degol, 2016). Classroom instruction limited by student attributes (CILSA) drives home the point that some factors such as weak foundational knowledge of poor nutrition, sleep problems, frequent absences, and behavioral problems may interfere with efficient teaching (OECD, 2019c). For example, hunger, absenteeism, fatigue, and a serious lack of previously learned skills in reading are just a few of the things that stand in the way of effective teaching, thus affecting SRA (Mullis et al., 2017). These factors often require teachers to make allowances by modifying instructional strategies in the form of lesson plans that accommodate unfocused students or frequently absent ones. Such adjustments, however, can easily disrupt lesson continuity, as well as reduce available time for the development of critical reading skills, thus making it difficult to maintain regular, quality instruction (OECD, 2018). Research shows that students with behavioral problems negatively affect the classroom atmosphere and that this situation reduces both teaching quality and academic achievement (McLeod and Kaiser, 2004).

### Profiling school academic success emphasis: effects on classroom interaction and student achievement

School academic emphasis has been conceptualized as a key institutional factor shaping the instructional climate and students’ academic outcomes, including reading. A substantial body of empirical research has provided considerable evidence to support the link between TEMS and SRA. Indeed, Göktaş and Kaya (2023) further support the finding that positive teacher-school community relations enhance the academic achievement of students. This finding suggests that when teachers build strong relationships within the school community, the resulting supportive environment reinforces their encouragement and motivation, ultimately leading to higher academic outcomes for students. For example, Reyes et al. (2012) state that students are able to show better academic achievement if they are more emotionally involved in the learning process. Indeed, emotionally supportive relationships at school provide such involvement. Similarly, findings by Bozkurt (2022) showed that teacher support is one of those aspects that has a profound impact on students’ reading performance.

The second topic covered by this study is how CILSA impacts SRA and thus highlights the need to understand the role of student engagement and individual student characteristics in teaching effectiveness. Scientific evidence from an earlier study by BouJaoude and Faour (2024a) showed that class limitations due to student characteristics, including unmet basic needs such as limited prerequisite skills and hunger, could hinder academic achievement. BouJaoude and Faour (2024a) findings further indicated that students facing these challenges are less engaged in learning activities, likely contributing to widening achievement gaps. This supports the understanding that reading outcomes and overall academic success require students to be prepared and to have their fundamental needs met. These insights are extended by Kalu and Ali (2004), who emphasize the positive contribution of specific student characteristics like prior knowledge and socio-economic status (SES) associated with positive learning outcomes. Similarly, Seidel’s (2006) research findings related to the same relationship reveal the means by which diverse student profiles influence perceptions of supporting learning environments and perceived instructional effectiveness.

Building on earlier studies, this research further examines the association between SEAS profiles and student reading achievement, investigating how these institutional priorities and support structures contribute to the literacy outcomes of these students. As will be shown, a recent study by Chechi et al. (2024) illustrates clearly that school emphases on comprehensive student competencies beyond traditional academics create environments that foster both academic and developmental success. Their findings indicate that initiatives focused on student readiness and wellbeing, for instance, the “Happiness Curriculum” indirectly promote engagement in school and achievement by underlining the importance of a school-wide focus on academic achievement. Indeed, BouJaoude and Faour (2024b) have illustrated that in schools that place a strong academic emphasis on attainment, environments are created which are conducive to SRA and other essential skill areas. Other findings by Dittrich and Staden (2025) show that schools with a focused academic emphasis register better literacy outcomes, likely due to improved motivation among the students in these schools. The impact of SEAS on students’ literacy skills has also been observed in international assessments. For example, studies such as PIRLS and PISA show that students’ literacy skills are generally higher in schools that place academic achievement at the center (Mullis et al., 2017; OECD, 2019b). However, whether this relationship is directly causal depends on various contextual factors. In particular, SES is a strong determinant of student achievement and can shape the impact of academic emphasis (Şirin, 2005). Therefore, in order to fully understand the relationship between academic emphasis and literacy achievement, variables such as school resources, teacher quality, and family support should also be taken into consideration (Hanushek and Woessmann, 2020).

## Conceptual framework

The guiding framework for this study adopts a school effectiveness perspective, integrating both traditional and broader definitions of how schools link to student learning. Consistent with classical approaches, student achievement is treated as the primary indicator of school effectiveness (Leithwood and Louis, 2021). However, previous research has increasingly emphasized that effectiveness cannot be understood through static, single-point observations alone. Broader definitions within educational effectiveness research highlight that student outcomes result from the interplay of multilevel school processes, including teaching, curriculum, and the learning environment (Creemers and Kyriakides, 2010; Reynolds et al., 2014). Methodological advances, particularly multilevel modeling, have further demonstrated that schools contribute meaningfully to variation in student outcomes by shaping these interconnected processes. Scholars have noted limitations in earlier effectiveness research for focusing heavily on identifying factors associated with student achievement while paying insufficient attention to the processes through which change and improvement occur (Muijs et al., 2014). Dynamic approaches argue that school effectiveness must be viewed as a developmental phenomenon, influenced by school contexts that evolve over time and by the capacity of schools to implement and sustain improvement practices (Creemers and Kyriakides, 2006; Scheerens, 2016).

The variable representing the context in this study is the importance given by teachers to academic achievement at the school level. The level of emphasis on academic achievement can play a role in increasing or limiting the impact of teaching processes. For example, in schools with a high emphasis on achievement, teachers may use more structured teaching methods and engage in strategic and consistent practices to increase student achievement. On the other hand, the effect of the same instructional efforts may be limited in schools with low emphasis (Burns and Stalker, 1994). Indeed, Hallinger and Heck (1996) stated that the relationship between school leadership and student achievement depends not only on administrative practices but also on the general academic atmosphere of the school. Similarly, Mulford and Silins (2003) emphasized that school environment and management structure are among the determinants of student achievement.

The research model developed in this context focuses on the relationship between teachers’ classroom practices and SRA. The two main instructional variables in the model are defined as TEMS and CILSA. While TEMS function more in psychological and interactional domains, CILSA function as factors that structurally affect the quality of instruction. The effect of these two variables on student reading achievement (SRA) may differ in line with the importance teachers attach to SEAS throughout the school.

**FIGURE 1:**
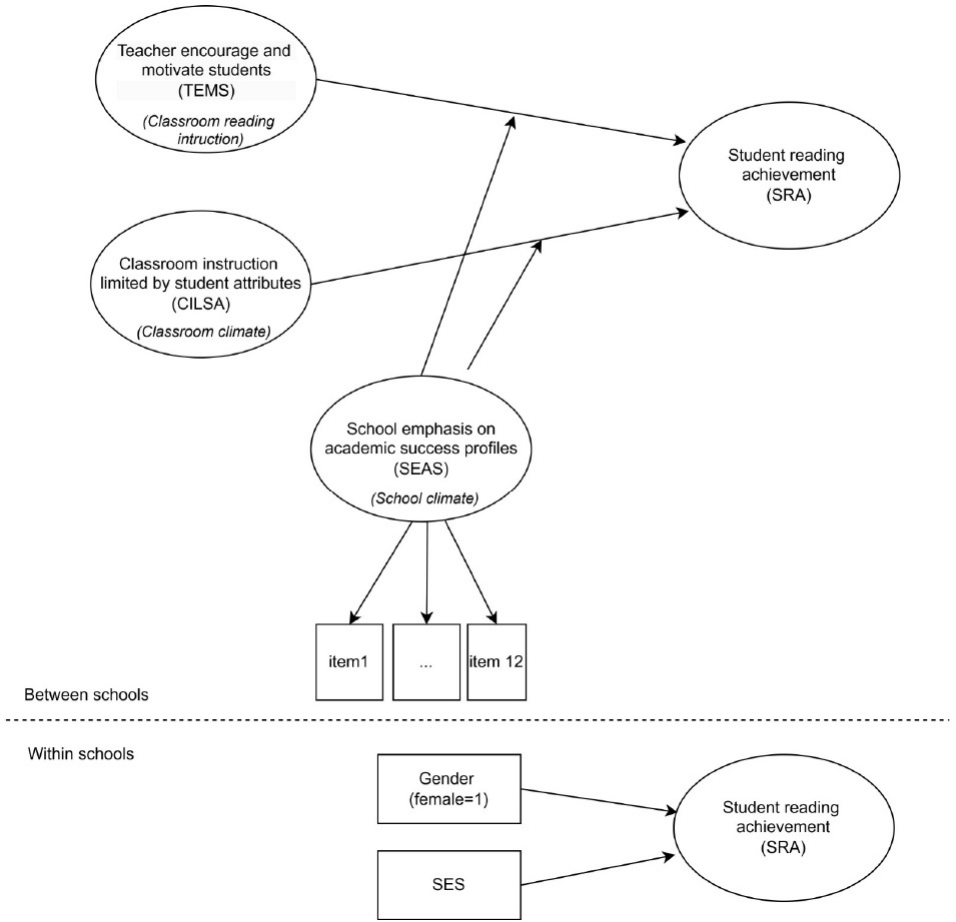
Study model.

In the research model, SEAS variable is a categorical variable representing two different emphases at the school level created by latent profile analysis (LPA). The model aims to reveal how SEAS profiles change the relationships between classroom practices and student outcomes. Therefore, this study conducted in the context of Türkiye, a non-European-American education system with its centralized and hierarchical structure, aims to contribute to the international literature at the theoretical and contextual level by examining the moderating effect of the importance given by the school to academic achievement on the relationship between teacher-based classroom practices and SRA. The research model is shown in Figure 1.

## Materials and methods

### Sample

This study utilized PIRLS 2021 data to analyze the reading achievements of fourth-grade students in Türkiye during the COVID-19 pandemic, incorporating perspectives from school principals, teachers, parents, and students. Employing a multi-stage cluster sampling method, the sample included 6,032 fourth-grade students from 192 schools, strategically selected to represent a broad cross-section of geographic regions and demographic characteristics as defined by the Statistical Institute of Türkiye. Initially, schools were clustered by geographic region and selected randomly. Subsequent random selection of fourth-grade classes from these schools ensured that all students in these classes were included in the sample. The diversity of the sample aimed to represent the national variance in reading skills, SES, school types, and geographical locations. The sample comprised 49.6% male students and 50.4% female students. The mean age of the participants was 9.97 (SD = 0.45).

### Measures

The study employed SRA, SES and three Likert-type scales: SEAS, TEMS, and CILSA, each measured on a 5-point scale ranging from very low to very high. The following section discusses the psychometric properties of these scales.

#### School emphasis on academic success

To measure SEAS, PIRLS conducted 12 items (ATBG10A-L). These items measure teacher responses on the degree to which students respect their classmates, parental expectations and support for student achievement, teacher expectations for student achievement, and collaboration among school principals and teachers. Examples of items include “Teachers’ expectations for student achievement” and “Parental commitment to ensure that students are ready to learn.” To assess the reliability of the scale, McDonald’s omega (ω) was used. The omega (ω) coefficient was calculated to be 0.91, indicating a high level of reliability. The examination of AVE results indicate consistency with a value of 0.78 and 0.76, supporting the construct’s validity. The results of the CFA revealed good fit indices [χ^2^/df = 125.00/46.00 = 2.72, RMSEA = 0.09 (90%CI, Lower = 0.08; Upper = 0.11), CFI = 0.95, TLI = 0.93, SRMR = 0.07].

#### Teachers encourage and motivate students

This scale consists of six items (ATBR09A-I) designed to evaluate how frequently teachers engage in activities to encourage and motivate students to read, such as providing materials suited to their reading level and encouraging students to challenge opinions. The reliability of the scale is reflected in McDonald’s omega (ω) coefficient of 0.88, indicating a high level of reliability. The results of the CFA revealed good fit indices [χ^2^/df = 39.30/11.00 = 3.57, RMSEA = 0.02 (90%CI, Lower = 0.01; Upper = 0.03), CFI = 0.99, TLI = 0.99, SRMR = 0.01].

#### Classroom instruction limited by student attributes

This scale consists of six items (ATBR03A-H) designed to assess the extent to which teachers believe that students in their classrooms face issues that limit instruction, such as lack of knowledge, sleep deprivation, mental impairment, or class absenteeism. The reliability of the scale is reflected in McDonald’s omega (ω) coefficient of 0.86, indicating a high level of reliability. The results of the CFA revealed good fit indices [χ^2^/df = 61.90/19.00 = 3.26, RMSEA = 0.09 (90%CI, Lower = 0.07; Upper = 0.12), CFI = 0.95, TLI = 0.92, SRMR = 0.04].

#### Student reading achievement

PIRLS generated five plausible values for overall reading (ASRREA01-ASRREA05). This study employed integration methods in Mplus software to use all values. SRA scores were calculated using five plausible values for each student provided by PIRLS. These values are used to represent SRA in a more reliable and statistically valid way. PIRLS calculates these five different plausible values to estimate the reading achievement level of each student. These values represent a wider range of SRA and provide a more accurate measurement with multiple values rather than a single score.

#### Student socio economic status

The SES index score of PIRLS 2021 was used in this study. The index consists of four items based on information provided by parents: (1) the total number of books in the home (0–10, 11–25, 26–100, 101–200, more than 200), (2) the number of children’s books (0–10, 11–25, 26–50, 51–100, more than 100), (3) the educational level of the parent (1: below primary school, 5: university and above), and (4) the occupational status of the parent (1: wage labor, 4: professional occupations). PIRLS combined these four variables to create a continuous SES score and categorized students into low (8.5 and below), middle (8.6–11.0) and high (11.1 and above) SES groups according to these scores.

### Analytical strategy

To identify schools with homogeneous subgroups of teachers based on their perceptions of SEAS, this study first employed LPA using standardized indicators from 12 items to address the first research question. LPA is a person-centered approach used to identify distinct school profiles. To decide on the number of profiles, this study tested models ranging from one to six latent profiles using 500 random starts and 100 final-stage optimizations. The optimal solution was based on multiple criteria, including the Bayesian Information Criterion (BIC), sample-size-adjusted Bayesian Information Criterion (aBIC), consistent Akaike Information Criterion (CAIC), and approximate weight of evidence criterion (AWE), which impose stronger penalties for model complexity and are therefore more conservative in selecting complex solutions (Bozdogan, 1987, 1993; McLachlan and Peel, 2000). Lower values of BIC, aBIC, CAIC, and AWE indicate better model fit, and the point at which these values are visualized on the elbow plot begins to flatten suggests the most parsimonious solution (Nylund et al., 2007). However, as the elbow plot showed relatively distinct patterns, this study also relied on likelihood-based tests [the bootstrapped likelihood ratio test (BLRT) and the Vuong–Lo–Mendell–Rubin corrected likelihood ratio test (VLMR)]. Significant *p*-values (*p* < 0.05) for BLRT and VLMR indicate that the k-class model fits the data significantly better than the k–1 class model (Nylund et al., 2007). The combined use of LPA and cross-level moderation moves beyond the uniform school climate assumptions of variable-centered models by identifying distinct school-level profiles and revealing how these profiles condition the effects of classroom practices across contexts. To address the second research question, this study employed a moderated effects model to examine how school profiles shape the relationship between classroom reading instruction, classroom climate, and SRA. To do this, a two-class latent profile solution was estimated at the school level, and a categorical latent variable representing each school’s most likely profile membership was incorporated directly into the mixture model rather than being entered as an observed variable. All predictors and outcomes were treated as observed variables and included in the model using their raw scores. Although the conceptual model illustrates these variables using oval shapes, they do not represent latent factors but rather observed indicators.

Next, plausible values (ASRREA01-ASRREA05) were included in the model using the multiple imputation (TYPE = IMPUTATION) framework in Mplus (Asparouhov and Muthén, 2010) to produce averaged parameter estimates with correct standard errors. Any missing values in the data were treated according to Mplus’s default missing data handling under the full information maximum likelihood (FIML) framework. Differences in slopes between the two latent profiles were tested using MODEL CONSTRAINT in Mplus. All analyses were conducted using Mplus Version 8.7 (Mutheìn and Mutheìn, 1998–2010). Model fit was evaluated using information criteria (AIC, BIC, SSABIC) and classification quality (entropy), and parameter differences between profiles were tested using a Wald test.

## Results

Table 1 shows the descriptive statistics and Pearson correlations between the variables at classroom level.

**TABLE 1:** Descriptive statistics and Pearson correlations between variables.

Variable	M	SD	S	K	SRA	Gen.	SES				
**Student level (*n* = 6,032)**											
1. SRA	504.30	84.80	−0.37	0.12	1.00	0.09**	0.47**				
2. Gender	49.6%	–	–	–		1.00	0.01				
3. SES	9.28	1.91	0.01	0.58							
**Variable**	**M**	**SD**	**S**	**K**	**ICC1**	**ICC2**	**SRA**	**SEAS**	**TEMS**	**CILSA**	**SES**
**School level (n = 192)**											
1. SRA	502.20	47.40	−0.53	0.49	0.27	0.86	1.00	0.22**	0.24**	−0.20**	0.80**
2. SEAS	9.75	2.44	0.70	1.48	–	–		1.00	0.04**	−0.04**	0.52**
3. TEMS	3.61	0.42	−0.65	1.58	0.07	0.65			1.00	−0.11**	−0.03
4. CILSA	2.40	0.82	−0.10	0.40	0.16	0.82				1.00	0.18*
5. SES	9.23	1.47	−0.09	0.32	0.39	0.94					1.00

*p < 0.05. **p < 0.01. M, Mean; SD, Standard deviation; S, Skewness; K, Kurtosis; SRA, Student reading achievement; Gender, Reference group is female; SES, Home socioeconomic status (ASBHSES); SEAS, School emphasis on academic success; TEMS, Teachers encourage and motivate students; CILSA, Classroom instruction limited by student attributes.

The mean scores indicated a high level of SRA among students and schools, with an average score of 504.30 and 502.20, respectively. A positive but small correlation was observed between gender, as represented by the percentage of female teachers, and SRA (*r* = 0.087, *p* < 0.01). A strong positive correlation was observed between SES and SRA (*r* = 0.47, *p* < 0.01) at the student level. This strong association between SES and SRA is largely expected in the Turkish educational context. In Türkiye, previous studies similarly have reported a strong relationship between students’ SES background and academic achievement (Ersan and Rodriguez, 2020; Özdemir et al., 2014; Özer and Suna, 2022). The present finding is therefore consistent with the existing literature, which consistently shows that SES remains one of the main factors underlying student achievement in Türkiye.

At the school level, a significant positive correlation was found between SEAS and SRA (*r* = 0.22, *p* < 0.01), and TEMS showed a moderate positive correlation with SRA (*r* = 0.24, *p* < 0.01). Conversely, CILSA showed a negative correlation with SRA (*r* = −0.20, *p* < 0.01). The correlation between SEAS and SES was found to be 0.52**.

To ascertain the suitability of aggregating teachers’ responses at the school level, the Intraclass Correlation Coefficients (ICC) were calculated. The ICC(1) values ranged from 0.07 to 0.27, while the ICC(2) values ranged from 0.65 to 0.86. As all ICC(1) values exceeded 0.05 and all ICC(2) values were above 0.40, it was appropriate to aggregate these variables at the school level.

### Identifying school profiles

The initial research question was: What distinct profiles of schools can be identified based on their emphasis on academic success, according to teacher perceptions? To explore this question, this study conducted an unconditional LPA, using the teacher responses on SEAS. Table 2 displays the fit statistics up to a six-profile solution.

**TABLE 2:** Fit Statistics for the LPA.

LP	Par	LL	BIC	aBIC	CAIC	AWE	BLRT	VLMR	Entropy	Lowest
1	24	−3218.00	6562.00	6486.00	6586.00	6759.00	–	–	–	193
**2**	**37**	−**2931.00**	**6057.00**	**5940.00**	**6094.00**	**6362.00**	**< 0.001**	**0.02**	**0.864**	**80**
3	50	−2768.00	5798.00	5640.00	5848.00	6210.00	< 0.001	0.09	0.850	22
4	63	−2684.00	5700,00	5500.00	5763.00	6219.00	< 0.001	0.35	0.801	11
5	76	−2596.00	5590.00	535.00	5666.00	6217.00	< 0.001	0.19	0.810	16
6	89	−2533.00	5533.00	5251.00	5622.00	6267.00	< 0.001	0.46	0.926	3

Bold values indicate the selected (best-fitting) LPA solution. LP, latent profile; Par, parameters; LL, log likelihood; BIC, Bayesian information criterion; aBIC, sample size adjusted BIC; CAIC, consistent Akaike information criterion; AWE, approximate weight of evidence criterion; BLRT, bootstrapped likelihood ratio test *p*-value; VLMR, Vuong-Lo-Mendell-Rubin adjusted likelihood ratio test *p*-value; cmPk, approximate correct model probability.

**FIGURE 2:**
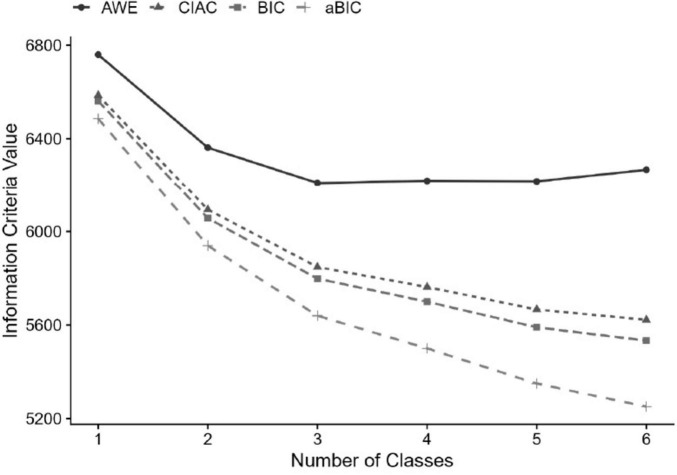
Elbow plots.

The results in Table 2 showed that the VLMR became non-significant after the third profile. However, in Figure 2, the information criteria indicated that the aBIC supported more than two profiles. A three-profile model also appeared plausible, given that the steepest decline in the AIC/BIC plots began to flatten after the third profile. Therefore, additional indicators were considered to determine the optimal number of profiles. First, although the information criteria showed a slight improvement when a third profile was added, the resulting additional class consisted of very few cases (*n* = 22) and exhibited low classification probabilities. Mutheìn and Mutheìn (1998–2010) emphasize that class sizes smaller than 50 cases do not allow for reliable generalization. Moreover, while the entropy value was 0.864 for the two-profile model, it decreased to 0.850 in the three-profile model. Taken together, these findings indicate that the substantive and statistical gains associated with adding a third profile were minimal, suggesting a plateau and a clear point of diminishing returns at the two-profile solution. In addition, an examination of the profile means showed that the three-profile solution did not introduce a theoretically distinct new class. Instead, it simply separated the existing low and high patterns observed in the two-profile model into a more graded structure, such as low, medium, and high. This refinement offered limited conceptual value and did not justify the increase in model complexity.

**FIGURE 3:**
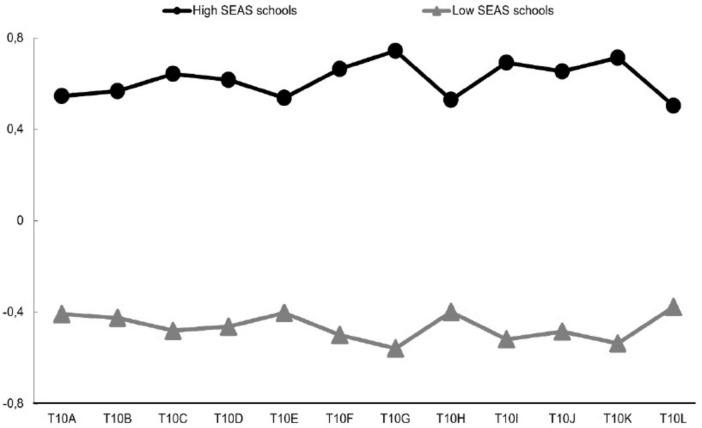
School profiles on school emphasis on academic success.

The characteristics of the school profiles are illustrated in Figure 3, which depicts the two distinct latent profiles. The first profile is characterized by a high perception of SEAS, with scores ranging from 0.50 to 0.74. This profile is referred to as “high SEAS schools” and reflects a high level of academic emphasis among school staff, parents, and students. It is estimated to comprise 42.60% (*n* = *81 schools*) of the study’s sample. In contrast, the second profile exhibits negative perceptions across all measures, with scores ranging from −0.38 to −0.56. This has led to it being termed “low SEAS schools.” This suggests minimal effort toward academic emphasis among school staff, parents, and students, with this group making up approximately 57.30% (*n* = *109 schools*) of the sample.

### Testing the moderation model

**FIGURE 4:**
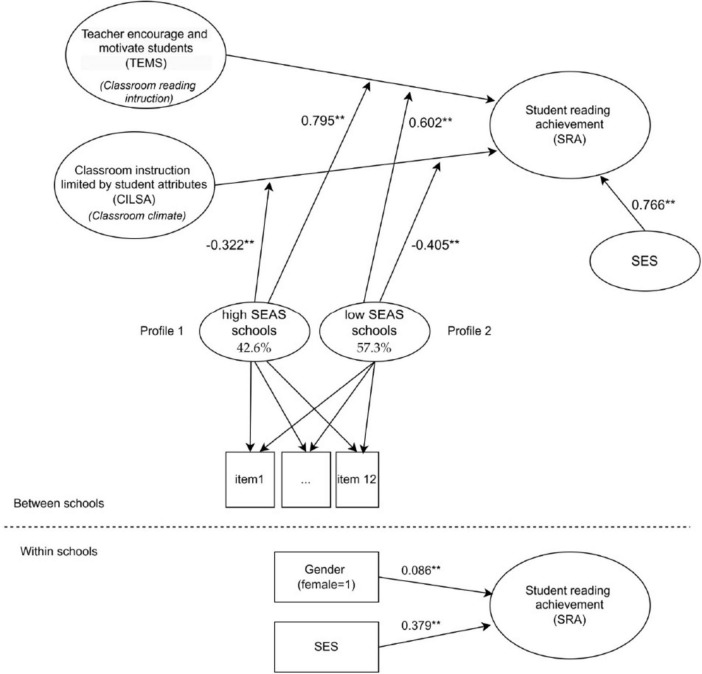
Model results. **p < 0.01.

In this step, the study tested the second research question, which was as follows: “Do school profiles moderate the relationship between classroom reading instruction, classroom climate, and student reading achievement?.” Model fit indices indicated an adequate fit of the two-class mixture solution (AIC = 67,688.51; BIC = 67,781.08; SSABIC = 67,736.59), with an entropy value of 0.995 suggesting excellent classification accuracy. A Wald test of equality constraints confirmed that the two profiles significantly differed in their overall reading performance levels (χ^2^ = 3.087, *p* = 0.0045). Figure 4 presents the model results.

**FIGURE 5:**
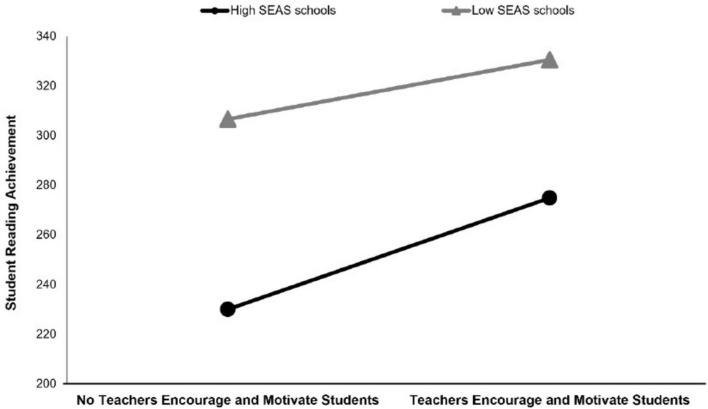
The moderating role of school profiles in the relationship between teachers’ encouragement motivation of students and student reading achievement.

Figure 4 indicated that for schools with a “high SEAS” profile, TEMS supported SRA with a positive effect [γ = 0.80, 95% CI = (0.70, 0.89), see Figure 5]. Also, Figure 4 indicated that SES affects the reading score by 0.77. This result indicated that the relationship between TEMS and SRA is reinforced in profiles where a school’s teachers perceive the SEAS to be higher. For schools with a “low SEAS” profile, there was an effect of TEMS on SRA [γ = 0.60, 95% CI = (0.50, 0.70)]. The difference in slope between the two profiles was notable, suggesting that schools with a “high SEAS” profile exerted a more pronounced influence on the correlation between TEMS and their SRA than those with a “low SEAS” profile [high SEAS- low SEAS = 20.99, 95% CI = (8.86, 33.14)]. Although the difference in slopes indicates a significant moderation effect, its magnitude is modest, suggesting that TEMS contributes to SRA in both school contexts, with only a limited additional benefit in high-SEAS schools.

**FIGURE 6:**
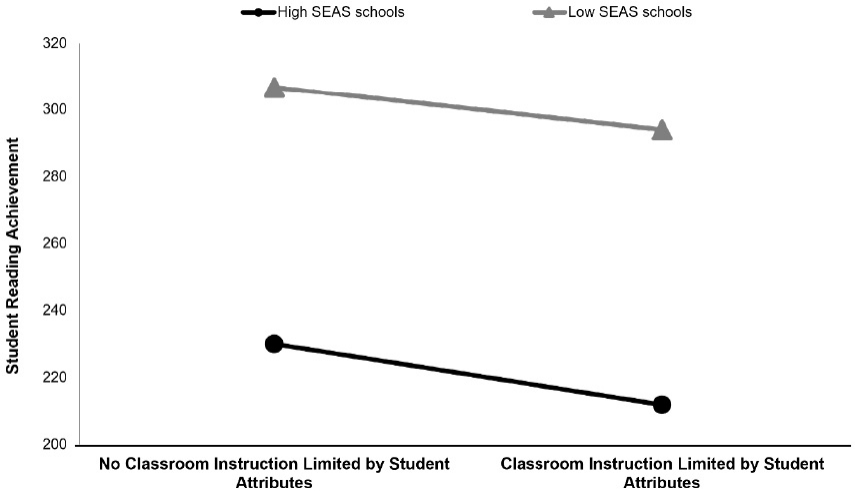
The moderating role of school profiles in the relationship between classroom instruction limited by student attributes and student reading achievement.

Additionally, the study findings indicated that for schools with a “high SEAS” profile, CILSA influenced SRA with a negative effect [γ = −0.32, 95% CI = (−0.42, −0.22), see Figure 6]. Also, for schools with a “low SEAS” profile, there was a negative effect of CILSA on SRA [γ = −0.32, 95% CI = (−0.41, −0.24)]. The difference in the slope between the two profiles was not significant, suggesting that there is no difference between the “high SEAS” and “low SEAS” profiles in terms of the correlation between CILSA and SRA [high SEAS- low SEAS = −5.36, 95% CI = (−10.93, 0.21)].

For the control variables, the model revealed a significant positive effect of student gender on achievement (β = 0.086, SE = 0.016, *p* < 0.001) and a strong positive effect of SES on achievement (β = 0.379, SE = 0.016, *p* < 0.001).

### Interpretation of findings

The first research question investigated whether schools were divided into different profiles based on the emphasis on academic success. Using multilevel LPA, this study found two main profiles, identifying schools with low and high emphasis on academic success. Schools with a high academic emphasis have factors such as high expectations for student achievement, teachers’ ability to inspire students, and parental involvement, while schools with a low academic emphasis have lower academic expectations, limited teacher interaction with students, and generally limited parental involvement. A possible explanation for the emergence of these two profiles is the factors that differ between schools, such as student profiles, parental involvement, teacher collaboration, and leadership practices. In Türkiye, students are generally assigned to schools according to their residential addresses, a practice that often results in the formation of relatively homogeneous school environments. This, in turn, may create significantly different opportunities compared with those available to children who attend schools in more affluent areas (Gegekoğlu, 2023). In their study on the mediating effect of parental involvement on student achievement in Türkiye, Özdemir et al. (2023a) found that increased parental support and engagement in school positively impacted student performance. On the other hand, low academic emphasis schools may indicate schools where parental involvement is limited, academic expectations are low, and teachers are less or not at all committed to student achievement. These research findings align with some recent studies (Erdem and Kaya, 2023; Liu et al., 2022). Contrary to recent studies suggesting a diminishing relationship between SES and academic achievement, Erdem and Kaya (2023) found that SES remains a significant determinant of academic performance in Türkiye. This situation may suggest a potential correlation between SES and academic achievement. Since the early studies, numerous research findings (Bourdieu and Passeron, 1990; Coleman et al., 1966; Husen, 1967; Şirin, 2005) have consistently supported the phenomenon that the environment in which students are raised often determines the educational outcomes. Comparable cross-national recent evidence from six Northern European countries also shows that the SES composition of a school is significantly and positively associated with reading achievement, even after controlling for students’ own background variables (Chzhen and Leesch, 2023).

The second research question was to investigate the moderating effect of school profiles on the relationship between classroom reading instruction, classroom climate and SRA. This study found a positive effect of TEMS on SRA in schools with a “high SEAS” profile. A similar study that supports this result reveals that in schools where student-teacher interaction is strong, students’ academic performance is high (Pianta et al., 2012). There is also evidence that classroom climate is one of the correlates of academic achievement (Erdem and Kaya, 2024). Recent studies in Europe also show that supportive school and classroom climates strengthen students’ positive academic emotions and intrinsic reading motivation, which in turn encourages the use of deeper learning strategies and higher reading performance (Dimitropoulou et al., 2025). And also, it can be stated that teachers’ encouraging and motivating approaches toward students play a crucial role in meeting the three innate needs; “competence,” “relatedness,” and “autonomy,” which are essential for individuals to achieve success (Deci et al., 1991). At the same time, East Asian research using PISA 2022 data suggests that hypercompetitive learning climates can exhibit non-linear or negative relationships with achievement and student wellbeing, and can be counterproductive when academic emphasis turns into pressure (Xu and Wen, 2025).

These findings are in line with school effectiveness research, which conceptualizes schools as multilevel systems in which school-level conditions, particularly academic emphasis, shape the extent to which classroom practices translate into student learning outcomes (Reynolds et al., 2014). In schools with a high SEAS profile, teachers’ incentives and motivation toward students are more effective, reflecting an alignment between academic emphasis and instructional strategies. On the other hand, the limited effect of the same strategies in schools with a low SEAS profile indicates a contextual mismatch.

The findings also show that CILSA has a negative effect on SRA in schools with both high SEAS and low SEAS profiles. A previous study supports this finding, highlighting that regardless of the quality of instructional practices, students struggle to learn effectively if their attention is diverted or they are disrupted by the misbehavior of other students (Gage et al., 2018). In schools with a high SEAS profile, factors such as high expectations for students, cooperation, and parental involvement in the school may have a positive effect on SRA, but limitations due to student characteristics may have suppressed these positive effects. In other words, no matter how high the importance schools attach to academic achievement, the fact that the teaching process is limited to student characteristics has negative consequences. Similarly, a comparable negative effect was observed in schools with a low SEAS profile. The fact that the slope difference between the two profiles was not significant (high SEAS—low SEAS) indicates that CILSA had a similar negative effect on SRA in both school profiles. The non-significance moderation effect of CILSA is consistent with this variable being largely determined by student-specific and relatively stable classroom constraints such as lack of readiness, behavioral problems, and absenteeism. The PIRLS framework also suggests that such microsystem characteristics directly constrain instruction and are weakly influenced by school-level cultural factors (Mullis and Martin, 2019). Similarly, BouJaoude and Faour (2024a) show that student-based structural barriers negatively impact instruction regardless of a school’s academic emphasis. Therefore, while SEAS profiles can strengthen teacher practices, they are limited in modifying the impact of these student-based constraints. A current body of research by Salas and Pascual (2023) offers supporting evidence for this finding, showing that while school SES impacts literacy development, student characteristics such as cognitive and linguistic skills play a significant role in determining SRA, regardless of the school’s SEAS profile?. These results show that even schools with high academic achievement expectations face similar problems in the face of limitations arising from student characteristics. In the light of these findings, it would be appropriate to emphasize that, despite the importance of collaboration and parental involvement, limitations due to student characteristics in the teaching process have a strong negative impact on academic achievement. This may point to the need for more effective pedagogical strategies and increased professional development opportunities, especially for teachers to cope with these constraints. In addition, it can be argued that there are situations that require more support and intervention in line with the importance SEAS places on success in schools, and that this can be strengthened through parental involvement and in-school co-operation.

These findings suggest that TEMS is particularly effective in schools with a stronger school culture oriented toward academic achievement. However, the fact that only 5% (0.05) of variance is explained suggests that these effects are significant but limited and have limited potential to create large-scale changes in the educational context. Such a level of explained variance aligns with previous research indicating that school and classroom level variables account for only a modest share of differences in student achievement, as students’ academic outcomes are predominantly shaped by SES and individual learner characteristics (Gustafsson et al., 2018; Şirin, 2005; Von Stumm, 2017). Thus, the practical relevance of these findings for school management and teacher education policies should be discussed, considering not only statistical significance but also the long-term effects of teachers’ motivational strategies.

One of the key contributions of this study is its use of a person-centered analytic approach to classify schools into distinct profiles. Whereas traditional variable-centered regression models assume a uniform relationship across all schools, the profile analysis employed here demonstrates that the effectiveness of teacher practices varies according to school context. This shows that the same instructional strategies can yield different outcomes depending on a school’s academic culture, highlighting the inherently context-sensitive nature of teaching effectiveness.

Finally, this study points to a strong and significant relationship between SES and SRA. In other words, SES plays a decisive role in students’ academic achievement and further research is needed to understand how this relationship is shaped by other environmental factors. Therefore, the impact of SES can be considered not only statistically significant reflected in its relatively strong coefficient (0.77) but also substantively meaningful as an indicator of broader structural conditions, such as school location, that shape students’ learning opportunities.

### Limitations and further research

Several limitations should be considered when interpreting the findings of this study. First, while the study analyzed a diverse sample of 6,032 students from 192 schools across various regions of Türkiye, the cross-sectional nature of the PIRLS 2021 dataset prevents us from drawing causal conclusions about the relationships between classroom reading instruction, classroom climate, and SRA. Future research utilizing longitudinal data could provide more insight into these relationships over time. Second, the study relies on self-reports from teachers, principals, and parents to assess key variables, which may introduce bias due to over- or under-reporting of practices and perceptions during the COVID-19 pandemic. Additionally, because several key variables were measured using teacher self-reports, the possibility of common-method variance cannot be fully ruled out, and future studies would benefit from incorporating multi-informant or observational measures. Collecting more objective measures or additional perspectives from students themselves could help address this issue. Third, although the sample represents various geographical and demographic characteristics, the findings may still be limited in generalizability, particularly to educational contexts outside of Türkiye, where cultural and systemic differences may play a significant role. The impact of SES profiles and geographical differences of schools in Türkiye on student achievement should be analyzed in more detail. In particular, the structural difficulties faced by students in schools with low SES may limit the effectiveness of teaching processes. Failure to meet the basic needs of students in these schools may negatively affect classroom learning processes and deepen inequalities in school achievement. Fourth, in this study, classroom climate was operationalized through two specific dimensions: TEMS and CILSA. While these indicators capture meaningful aspects of classroom climate, they do not encompass the full multidimensional nature of the construct. Accordingly, the findings should be interpreted with the understanding that they reflect only the dimensions measured in this study rather than the entire spectrum of classroom climate components. Fifth, due to the LPA approach used in this study, profile memberships are probabilistic and interpreted based on the “most likely profile” designation. Therefore, there is a low probability of misclassification during the classification process, and the findings should be interpreted with this possibility in mind.

Finally, reapplying the model used in this study to PIRLS data from different countries is, as well as to subsequent PIRLS cycles, is important to test the extent to which the findings hold true in cross-border contexts. Using the same analytical approach across education systems with different governance models centralized, semi-centralized, or more decentralized structures will shed light on how the relationships between school profiles and student achievement vary by context.

## Conclusion

This study has revealed important findings regarding the contextual nature of teaching effectiveness by demonstrating how teachers’ emphasis on academic success at the school level shapes the relationships between classroom teaching processes and student achievement. The two distinct school profiles obtained high and low academic emphasis demonstrate that the impact of teacher behavior on students is influenced not only by teaching strategies but also by the school’s shared culture of success. Moreover, teacher motivation in schools with a strong academic focus is associated with higher reading achievement among students. This finding demonstrates that instructional effectiveness depends not only on pedagogical interventions but also on the shared values and collective expectations of the school, supporting the central proposition of school effectiveness research that school academic culture shapes the effectiveness of classroom practices (Muijs et al., 2014; Reynolds et al., 2014). In contrast, CILSA leads to similar negative effects in both school profiles, suggesting that structural inequalities can limit the effectiveness of teaching strategies. The findings indicate that the school’s academic vision must be collectively constructed in order for teachers’ motivational efforts to be more effective. However, since this effect can only explain a small portion of the variance in student achievement, multi-layered education policies that overcome structural barriers are needed. In this regard, the practically modest magnitude of these effects should be acknowledged, and policies aimed at enhancing teacher practices should be developed in conjunction with more dominant determinants of student achievement, such as SES and individual learner characteristics. Sustainable learning environments can be created by supporting schools with low academic emphasis, increasing teacher capacity, and reducing student inequalities. In this context, rebuilding school culture in centralized systems is only possible through a comprehensive approach that encompasses not only legislation but also teacher training, professional development and school leadership practices. In this respect, the study makes important contributions to both context-based education research and school development policies.

These findings offer some practical recommendations for schools and policymakers. First, leadership and collaboration programs aimed at strengthening school culture should be supported in schools with low academic emphasis. Second, targeted professional development programs should be established to strengthen teacher practices that enhance student motivation. Finally, providing early academic support and additional resources to disadvantaged schools is crucial to mitigate structural constraints stemming from student characteristics.

## Data Availability

Publicly available datasets were analyzed in this study. This data can be found at: https://pirls2021.org/data/.
